# Toxic Stress as a Potential Factor Inducing Negative Emotions in Parents of Newborns and Infants with Cyanotic Congenital Heart Disease

**DOI:** 10.3390/children10121893

**Published:** 2023-12-06

**Authors:** Grażyna Cepuch, Agnieszka Kruszecka-Krówka, Anna Lalik, Agnieszka Micek

**Affiliations:** 1 Nursing and Midwifery Institute, Faculty of Health Sciences, Jagiellonian University Medical College, 31-501 Krakow, Poland; grazyna.cepuch@uj.edu.pl (G.C.); anna.lalik@uj.edu.pl (A.L.); 2 Statistical Laboratory, Faculty of Health Sciences, Jagiellonian University Medical College, 31-501 Krakow, Poland; agnieszka.micek@uj.edu.pl

**Keywords:** infants, cyanotic heart defect, parents, negative emotions, toxic stress

## Abstract

Background: Parents who have a newborn with a congenital heart defect experience negative emotions, which may determine the emotional state of their children. Methods: The study group included 154 parents of newborns and infants with cyanotic congenital heart disease, before cardiac surgery and after the procedure. HADS m and PSS-10 questionnaires were used to assess parental anxiety, depression, aggression, and the level of stress. Results: High levels of depression, anxiety, total HADS and stress were diagnosed in a large group of parents, regardless of the stage of cardiac surgery treatment. A high level of stress was associated with a higher prevalence of emotional disturbance both in the total HADS (overall) and in all its individual domains. Anxiety and depression were more common in mothers. A high level of stress was a significant predictor of anxiety and depression in parents. Conclusions: A high level of stress was a significant predictor of anxiety and depression in parents of infants with congenital heart disease. The parents’ psychological condition is one of many potential determinants over the course of their child’s treatment and recovery.

## 1. Introduction

About 400,000 babies are born in Poland every year. In 2021, more than 56 infants per 100,000 live births died due to congenital disorders (in accordance with the International Statistical Classification of Diseases and Related Health Problems—ICD-10 Revision). The main cause of death tended to be cardiac disorders—42 per 100,000 live births [1] just like in other countries [2]. In 2019, 2020 and 2021 in the group of newborns and infants, the number of cardiac surgeries performed was, respectively, 882,858, and 919 based on the National Registry of Cardiac Surgeries—KROK. However, in Poland, the inclusion of pediatric cardiology into the list of priority specialties can be considered one of the important achievements in this area (the decision of the Polish Ministry of Health; Journal of Law of 23 September 2018, position number 1738) [3].

Despite optimistic reports on the survival of newborns and infants with congenital heart disease, parents confronting their child’s illness and the threat of death are likely to experience a wide range of negative emotions [4,5,6]. As indicated by scientific reports in this area, a significant percentage of parents experience post-traumatic stress and more than half of them present symptoms of depression and anxiety [7]. Mothers, in particular, are exposed to emotional burdens [4,8,9], which may result from the social roles assigned to them and their more frequent presence with the child during hospital treatment. Such persistent negative psychological experiences in parents can be observed not only at the stage of diagnosis and after cardiac surgery, but also during the subsequent developmental stages of the child [10]. High levels of stress, anxiety and depression (understood as a broad spectrum from emotional disorders of a depressive nature to clinical depression) affect both the mental health of the parents themselves and the parent–child relationship, which determines the child’s holistic development [9,11,12,13]. To a large extent, parents’ emotional condition may affect the child’s treatment and recovery process. Therefore, the knowledge of the nature of stress should enable the medical team to recognize typical symptoms of stress more quickly [14] and take measures to support parents [15].

It is vital to take into account parents’ emotional condition during their child’s multidisciplinary treatment, not only because it can be beneficial in terms of the time which the child stays in hospital, but also in terms of the future of the entire family. In view of the scarcity of reports in this area involving parents of newborns and infants with congenital cyanotic heart disease, at various stages of defect diagnosis (prenatal vs. postnatal period) and cardiac surgery treatment, an attempt has been made to assess levels of anxiety and depression, as well as perceived stress, using tools of a screening nature. This will also allow other members of the treatment team, not only psychologists, to recognize parental disorders as early as possible. Implementing measures aimed not only at the child, but also at the parent, will ensure optimization of care, making it more individualized, more relevant and more sensitive.

The aim of the study was to assess the prevalence of anxiety, depression, aggression/irritability and levels of perceived stress in a group of parents of newborns and infants with cyanotic heart defects, before cardiac surgery and after the procedure.

## 2. Materials and Methods

### 2.1. Study Design

The cross-sectional study was designed to evaluate the prevalence of anxiety, depression and aggression in parents of children diagnosed with cyanotic congenital heart disease, as well as to assess their stress levels. We used the STROBE checklist for cross-sectional studies when writing our report [16]. The study was carried out in a group of parents who exercised parental care over their child during the child’s hospital stay. The privacy and confidentiality of participants was strictly protected. All the information provided by each participant was coded by a number that does not directly identify any individual and all identifying information was coded and removed from all non-numerical data to make it impossible for anyone but the experimenter to identify any individual. The study was carried out in accordance with the ethical principles of the Helsinki Declaration. The protocol of the study was approved by the Bioethics Committee of the Jagiellonian University (No. 118.6120.86.2023)

In order to achieve the assumed goal of the study, the following research questions were formulated:

1. What are the levels of anxiety, depression and aggression in mothers and fathers of children with cyanotic heart disease depending on the stage of the patient’s cardiac surgery treatment?
2. What is the level of perceived stress in mothers and fathers of children with cyanotic heart disease depending on the stage of the patient’s cardiac surgery treatment?
3. What is the relationship between the negative emotions studied and the level of stress, and selected sociodemographic variables, the stage of treatment and the time of diagnosis of the defect in the child?

### 2.2. Sample, Setting & Data Collection

The study group included 154 parents. The study was conducted among parents of newborns and infants with cyanotic congenital heart disease at the Children’s Cardiology and Cardiac Surgery Center in Southern Poland from 10 January 2023 to 20 May 2023.

The process of recruiting parents for the study was carried out in two stages. In the first stage of group selection, the medical records of the cardiology and cardiac surgery departments were used, on the basis of which only parents of children aged 0 to 12 months with a diagnosed congenital cyanotic heart defect were pre-selected. The defects included: tetralogy of Fallot—TOF, transposition of the great arteries—TGA, hypoplastic left heart syndrome—HLHS, truncus arteriosus communis—TAC, tricuspid atresia—TA, pulmonary atresia—PA, and total abnormal pulmonary venous return—TAPVR. The children were in stable health (without radical deterioration due to the nature of the disease and the treatment used), not burdened by other conditions, and at various stages of cardiac surgery treatment (before or after defect correction). Correction of the heart defect was complete or incomplete, and incomplete correction of the heart defect meant one of the other stages of cardiac surgery treatment. Then, from the group of parents selected in the first stage of recruitment, only those who met further criteria were chosen for the study. These criteria included: not having another/other chronically ill or congenitally defective children and declaring to be a full partner or married family. Parents who reported a traumatic situation in the family during the child’s illness were excluded from the study. Situations considered as traumatic were serious illness or death in the family, planned separation or divorce, loss of job or other situation declared by the respondent as difficult.

The research material was collected during direct meetings with parents of hospitalized children. Participation in the study was voluntary and anonymous. Each parent received written and oral information about the purpose of the study and the possibility of resigning from participation in the study at any stage, without giving a reason and without bearing any consequences. Parents received information about the planned publication of the study results and all of them agreed.

Statistical analysis was performed taking into account only the questionnaires in which all tool sheets were fully completed.

### 2.3. Participants and Public Involvement

The study examined the patients’ parents, who were the leading source of information. The patients themselves were not involved in the study in any way.

### 2.4. Description of Research Tools

To assess parental anxiety, depression, aggression, the level of stress the method of diagnostic survey was applied including the following survey questionnaires:

- Self-designed questionnaire—regarding sociodemographic data: respondents’ age and gender, education, place of residence, child’s age, type of disease and its stage of treatment (e.g.: before cardiac surgery, after cardiac surgery).
- The Hospital Anxiety and Depression Scale (HADS), developed by Zigmond and Snaith [17], in the Polish adaptation by Majkowicz et al. [18]. It consists of three subscales assessing the occurrence of anxiety, depression and aggression/irritability among psychiatric and non-psychiatric patients who require assessment of their emotional state. Aggression examined with this tool should be understood as a state of emotional irritation or a feeling of aggression. The subscales can be interpreted separately or in total. The HADS scale was used due to its advantages, such as general availability, ease of use, possibility of use by nurses and doctors, and no need to involve psychologists, which significantly reduces the costs of using it. The analysis of the respondents’ answers was made in accordance with the instructions of the authors of the scale and the authors of the adaptation [17,18]: (i) no disorders: 0–7 pts.—depression/anxiety subscale, 0–2 pts.—aggression/irritability subscale; (ii) borderline states: 8–10 pts.—depression/anxiety subscale, 3 pts.—aggression subscale; (iii) observed disorders: 11–21 pts.—depression/anxiety subscale, 4–6 pts.—aggression subscale. For the purposes of the current study, each subscale was analyzed separately as well as in total.
- The Perceived Stress Scale—10 (PSS-10), developed by Cohen et al. [19], in the Polish adaptation by Juczyński and Ogińska-Bulik [20]. A screening scale is used to identify people requiring psychological support. The analysis of respondents’ answers and the calculation of results were consistent with the key developed by the authors of the tool and the authors of the adaptation [19,20]. Therefore, a result of 1 to 4 sten was considered as low, 5 to 6 sten was considered as average, and 7 to 10 sten was considered as high. A high score on the PSS scale is an indicator of the assessment of one’s own life situation as stressful and excessively burdensome.

### 2.5. Statistical Analysis

All categorical variables were presented by counts and percentages. For the purposes of the analysis, according to the definition in section: “2.4. Description of research tools”, the total HADS and its three domains (depression, anxiety and aggression) were dichotomized into two groups: 1—no disorders/borderline states, and 2—disorders. Scores from the PSS-10 questionnaire were grouped similarly. Scores classified as low/medium (average) were combined to form group 1—low; high scores constituted group 2—high. Differences in the distribution of HADS between PSS categories, socio-demographic features and variables characterizing the disease were assessed using chi-square test of independence. In the multivariable analysis the following covariates were used: gender (male, female), parents’ age (≤29, 30–34, >34 years), place of residence (city, village), time of diagnosing heart defect in the child (prenatal, postnatal), education (higher, other), correction of heart defect (complete, incomplete, none).

For each dichotomized HADS separately as a dependent variable (total HADS as well as depression, anxiety and aggression), multivariable logistic regression analysis was performed to check the contribution of PSS and covariates to HADS disorders. Odds ratios (ORs) with 95% confidence intervals (CIs) were extracted from the models. The statistical analyses were performed using the R Software for Windows (R Foundation for Statistical Computing, Vienna, Austria, version 4.0.4). Two-tailed significance level was set at *p* < 0.05.

## 3. Results

### 3.1. Characteristics of the Study Group

The groups of parents aged up to 29, 30–34 and over 34 were in similar numbers. The vast majority of respondents were women (85.71%) and parents living in cities (77.27%). More than half of the respondents had a university education. Complete correction of the heart defect was observed in 15.58% of newborns and infants, and 53.25% of children had an incomplete correction of the defect performed. More than 72% of newborns and infants were diagnosed with the disease prenatally. High levels of depression, anxiety and total HADS were diagnosed in 41.56%, 68.18% and 66.23% of parents, respectively, while borderline states of anxiety and depression and total HADS were observed in 34.42%, 24.03% and 24.68% of respondents, respectively. In 75.97% of parents, stress level was classified as high (Table 1).

### 3.2. Anxiety, Depression and Aggression/Irritability Levels vs. Parental Stress Level and Selected Socio-Demographic and Clinical Variables

A high level of stress, assessed using the PSS-10, was associated with a higher prevalence of emotional disturbance both in the total HADS (overall) and in all its individual domains (depression, anxiety, aggression). In addition, the results of the analysis more often indicated the presence of aggression/irritability in the youngest parents (up to 29 years old), while depression was twice as often present in urban residents. The respondents’ gender, education, stage of cardiac surgery treatment and the time of the disease diagnosis had no effect on the prevalence of emotional disorders assessed using the HADS scale—Table 2.

### 3.3. Probability of the Incidence of Anxiety, Depression and Aggression/Irritability Depending on Parents’ Stress Levels

Regardless of the selected variables, parents experiencing high levels of stress were significantly more likely to suffer from emotional disorders, ranging from a 3.12 times higher probability for the anxiety domain to a 13.75 times higher probability for the total HADS, compared to parents with low or moderate level of stress (Table 3). Parents aged 30–34 and ≥35 were, respectively, 64% and 54% less likely to experience aggression compared to parents under 29. Parents living in the city were more likely to suffer from depression and total HADS disorders compared to parents living in rural areas (OR = 2.81, 95% CI: 1.13; 7.01 for depression and OR = 2.57, 95% CI: 1.00; 6.55 for total HADS). Parent’s education and the stage of cardiac treatment did not affect the risk of depressive disorders, anxiety and aggression. Parents of children diagnosed with a heart defect postnatally were about 52% and 64% less likely to suffer from depression and total HADS, respectively, compared with parents of children diagnosed with a heart defect prenatally (OR = 0.48, 95% CI: 0.21, 1.13 for depression and OR = 0.36, 95% CI: 0.14, 0.90 for total HADS). However, this probability lost significance after additional adjustment for variables such as education and the stage of cardiac surgery treatment for the heart defect.

### 3.4. The Assessment of the Relationship between PSS-10 Score and HADS Domains as Continuous Variables

**Figure 1:**
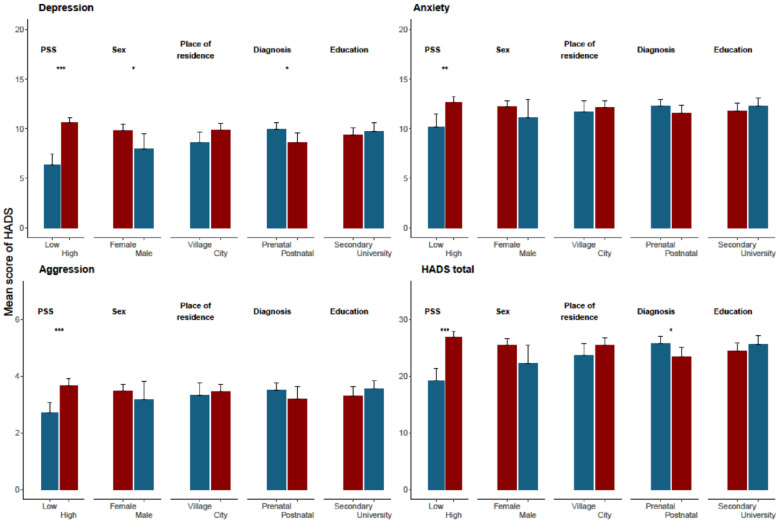
The comparison of HADS, PSS-10 scores and other covariates. *** *p* < 0.001, ** *p* < 0.01, * *p* < 0.05.

In addition, the relationship between the PSS-10 score and the HADS domains as continuous variables was tested. Pearson correlation coefficients oscillated from 0.331 and 0.336 for anxiety and aggression to 0.547 and 0.591 for HADS in total and depression, respectively, showing significant (*p* < 0.001) associations of weak-to-moderate strength (Figure 1).

Compared to parents with low stress levels, those with a high stress level had significantly higher scores (as continuous) in all HADS domains: depression (Mean = 10.6, SD = 2.92 vs. Mean = 6.32, SD = 3.59), anxiety (Mean = 12.69, SD = 2.93 vs. Mean = 10.14, SD = 4.14), aggression (Mean = 3.67, SD = 1.33 vs. Mean = 2.70, SD 1.18) and HADS in total (Mean = 26.96, SD = 5.49 vs. Mean = 19.16, SD = 7.13). Additionally, a prenatal diagnosis of a heart defect was associated with higher depression and HADS total scores compared with postnatal diagnosis, as well as female parents manifesting enhanced depression scores compared with fathers (Figure 1).

## 4. Discussion

A diagnosis of a heart defect in a child can generate a spectrum of negative emotions in parents, including post-traumatic stress. Parents’ psychological condition is one of many potential determinants over the course of their child’s treatment and recovery, both before and after cardiac surgery. It is also important in terms of future development and behavioral outcomes in pediatric patients with congenital heart defects [6,8,9,12,21,22]. A report by Ribaudo [23] indicates a close synchronization of parent–infant interactions. Parents who struggle with high levels of stress may have greater difficulties in their relationship with their child, which increases the risk of children developing social and emotional problems later in life.

The current study aimed to assess the prevalence of anxiety, depression and aggression/irritability, as well as the level of perceived stress in parents of newborns and infants with cyanotic heart defects, before and after cardiac surgery.

The results of the study showed that having a child with a cyanotic congenital heart defect induced high levels of stress as well as depressive and anxiety disorders in the vast majority of parents. It also indicated a correlation between high levels of stress and the incidence of negative emotions. Anxiety and depression were more common in mothers and they were the primary caregivers for the child during hospitalization, similarly to the reports by other authors [4,8]. Higher levels of negative emotions in mothers may have been determined not only by individual gender-related resources, but also by the burden of assuming the role of primary caregiver during hospitalization, which in many cultures [24,25], including Polish [26], still remains the domain of women. At the same time, mothers who become an important part of the treatment team are more likely to be emotionally and physically overburdened than fathers [27]. The father, according to the still functioning division of social roles, separate for men and women, is sometimes seen as a family member with a different approach and view of the child’s illness than the mother [4,11]. However, this model is gradually changing [28,29] and fathers seem to be more and more involved in their child’s care [30]. Although mothers were more likely to experience high levels of anxiety and depression, it should be noted that fathers also suffered from disorders in the emotions studied. Fathers experience many demands and stressors related to their child’s congenital heart disease. The stress they experience is related to the inability to protect the child from the consequences of illness, hospital stay and treatment process, but also to difficulties in reconciling professional duties and support for the partner [11]. They also experience stress in the absence of targeted interventions that they expect and that would meet their needs [29].

Regardless of the stage of the child’s cardiac surgery treatment, the dominant emotion in the study group was anxiety, which affected parents of both sexes. Respondents also experienced aggression/irritability, which was present both in mothers and fathers. It is worth noting that the diagnosis of congenital cyanotic heart defect, as well as the performed correction of the defect, complete or incomplete, does not give certainty about the further health and developmental prognosis of the patients. Anxiety and emotional distress may accompany parents at every stage of their child’s treatment and life [31,32]. However, a report by Składzień et al. [33] showed significantly lower levels of anxiety in parents of children after cardiac surgery than in a group of mothers and fathers of children before defect correction. The differences obtained may result from different criteria for the selection of the study group. Nevertheless, it should be taken into account that the level of negative emotions, including anxiety, may be high in children’s parents both before and after cardiac surgery.

The study showed no significant differences between anxiety and aggression/irritability depending on the time of the child’s heart defect diagnosis (prenatal/postnatal period), no differences in stress levels, even though the percentage of parents with these disorders was high. However, younger parents showed significantly higher levels of aggression/irritability. It cannot be ruled out that the reason for this reaction was not only the diagnosis of a heart defect in their child, but also the experience of adjusting to a new role as a parent, as well as personal resources, other than aggression/irritability, to cope with stress.

The current study indicated the presence of depressive disorders in a significant percentage of respondents, with mothers scoring statistically significantly higher on the depression scale compared to fathers. The incidence of depression was correlated with the place of residence and the time of defect diagnosis. Parents of children diagnosed with the defect prenatally and living in the city were more likely to develop depressive disorders. Perhaps the high level of depression in parents of children diagnosed with a heart defect in the prenatal period was related to a prolonged sense of threat to their child’s health and life and uncertainty about the course of pregnancy, childbirth and further prognosis. Similar results regarding the percentage of parents with diagnosed depression before their child’s heart defect was corrected were obtained by other authors [32,33], but the number of respondents who were diagnosed with depression in the period after the cardiac surgery was significantly lower [33] or comparable to results of the current study [34].

Based on the results obtained in the study, as well as on an analysis of scientific reports in this area, it can be concluded that parents of infants with congenital heart defects, especially mothers [35,36,37] may suffer not only from anxiety, depression and aggression/irritability, but also from high levels of stress. It cannot be ruled out that, among many potential causes, the reason for the high percentage of parents with high levels of stress and negative emotions was the inadequate support provided to them by interdisciplinary teams. Cyanotic heart defects are considered to be conditions with a difficult-to-predict trajectory of consequences, so they can generate negative emotional tension [31].

The results of scientific studies [13,33,37,38,39,40], as well as the current study, indicate high levels of stress in parents of children with congenital heart defects regardless of the stage of cardiac surgery treatment, despite the fact that the cited authors used different measurement tools. The high prevalence of stress among parents of children with congenital heart defects is related not only to the child’s illnesses experienced [9] and the parent–child system, but also to a number of other parental, environmental and hospital factors [41], as well as dysfunctions within their own social network. Recognizing parents with emotional disturbances and high levels of stress can be crucial not only for themselves, but also for their child [42].

### 4.1. Implications

The results obtained are in line with other research in the area of family functioning and emotions of parents of children with congenital cyanotic heart defect, proving that the diagnosis of a defect in a child, regardless of the stage of treatment, provokes severe stress and generates negative emotions in parents. The survey tools applied are generally available, free and easy to interpret. They can be used by nurses or doctors as good screening tools. Preliminary assessment of disorders by members of medical teams who are not psychologists can accelerate the identification of parents in need of a prompt professional intervention. What is urgently needed is appropriate curricula, extended psychological support and informational materials [42]. Particular attention should be paid to making medical teams more sensitive to the incidence of stress, including toxic stress and stress-related emotional disorders in parents of children with cyanotic heart defects. This will make it possible to quickly and efficiently provide specialized (psychological and psychiatric) assistance to parents of sick children at every stage of hospitalization, which may also contribute to reducing social costs.

### 4.2. Strengths and Limitation

The study conducted has a number of limitations. Firstly, it was conducted in only one center in southern Poland, and the homogeneous group of participants represents only a slice of the population of parents taking care of their children with this type of defect. A much larger number of mothers than fathers participated in the study, which may have influenced the lack of statistically significant differences between the sexes. The lack of equality of the groups may be the reason for the underestimation of the results in the male group.

Further research is needed, also in a group of fathers, to understand the importance of emotional experiences and be able to develop interventions targeting both parents. In the study presented here, fathers were in a distinct minority, which may have affected the results. Focusing also on fathers in the diagnosis of emotional disorders and stress, may help to change their role in the child’s treatment and reduce feelings of helplessness, both in the father and the mother.

## 5. Conclusions

Negative emotions accompany most of mothers and fathers of newborns and infants with cyanotic congenital heart disease, regardless of the stage of cardiac surgery treatment and the high level of stress is an important predictor of the mental state of the parents. However, in light of the findings of the current study, there are some groups of parents especially vulnerable to negative feelings who may require specific psychological help. It was shown that mothers and those who had heard the diagnosis of a heart defect in their child in the prenatal period might be at a higher risk of depression and should be a target population of psychological support in this area. On the other hand, younger parents of ill children have a tendency to be more aggressive, so psychological care can be aimed, to a larger extent, at coping with these emotions.

### Search Strategy and Selection

The choice of research strategy was aimed at assessing the impact of negative emotions, stress, coping strategies and congenital heart defect. The report was prepared following STROBE guidelines and English language articles published between 2010 and 2022 and available in Google Scholar and PubMed databases. The search was completed in December 2022. The keywords applied in the search included parents, congenital heart defect in newborns and infants, depression, aggression, anxiety, coping strategies, mental disorders.

## Figures and Tables

**Table 1:** Characteristics of the group of parents and their children diagnosed with cyanotic congenital heart disease and basic features of detected defects in children (N = 154).

Variable	Categories	n (%)
Gender	female	132 (85.71)
	male	22 (14.29)
Age	≤29	51 (33.12)
	30–34	53 (34.42)
	≥35	50 (32.47)
Place of residence	>500,000	26 (16.88)
	200–500,000	30 (19.48)
	100–200,000	35 (22.73)
	<100,000	28 (18.18)
	village	35 (22.73)
Education	elementary	2 (1.30)
	vocational	18 (11.69)
	secondary	54 (35.06)
	high	80 (51.95)
Time of disease diagnosis:	prenatal	111 (72.08)
	postnatal	43 (27.92)
Type of disease	TOF	42 (27.27)
	TGA	23 (14.94)
	HLHS	39 (25.32)
	TA	12 (7.79)
	TAC	14 (9.09)
	PA	12 (7.79)
	TAPVR	10 (6.49)
	other cyanotic defects	2 (1.30)
Defect correction	complete	24 (15.58)
	incomplete	82 (53.25)
	none	48 (31.17)
HADS Depression—categories	no disorder	37 (24.03)
	borderline	53 (34.42)
	disorder	64 (41.56)
HADS Anxiety—categories	no disorder	12 (7.79)
	borderline	37 (24.03)
	disorder	105 (68.18)
HADS Aggression/Irritability—categories	≤3	80 (51.95)
	≥4	74 (48.05)
HADS total	no disorder	14 (9.09)
	borderline	38 (24.68)
	disorder	102 (66.23)
PSS-10 catergories	low	3 (1.95)
	average	34 (22.08)
	high	117 (75.97)

**Table 2:** Frequency of occurrence of HADS disorders according to basic characteristics of children and their parents (N = 154).

Variable	Categories	Depression			Anxiety			Aggression/ Irritability			Total		
		None + Borderline	Disorder	*p*	None + Borderline	Disorder	*p*	≤3 pkt	≥4 pkt	*p*	None + Borderline	Disorder	*p*
Gender	female	74 (56.06)	58 (43.94)	0.217	39 (29.55)	93 (70.45)	0.216	67 (50.76)	65 (49.24)	0.621	42 (31.82)	90 (68.18)	0.313
	male	16 (72.73)	6 (27.27)		10 (45.45)	12 (54.55)		13 (59.09)	9 (40.91)		10 (45.45)	12 (54.55)	
Place of residence	village	26 (74.29)	9 (25.71)	0.049	12 (34.29)	23 (65.71)	0.881	16 (45.71)	19 (54.29)	0.517	17 (48.57)	18 (51.43)	0.057
	city	64 (53.78)	55 (46.22)		37 (31.09)	82 (68.91)		64 (53.78)	55 (46.22)		35 (29.41)	84 (70.59)	
Education	other	46 (62.16)	28 (37.84)	0.461	25 (33.78)	49 (66.22)	0.741	43 (58.11)	31 (41.89)	0.190	26 (35.14)	48 (64.86)	0.861
	higher	44 (55.00)	36 (45.00)		24 (30.00)	56 (70.00)		37 (46.25)	43 (53.75)		26 (32.50)	54 (67.50)	
Time of diagnosis	prenatal	60 (54.05)	51 (45.95)	0.111	35 (31.53)	76 (68.47)	1.000	58 (52.25)	53 (47.75)	1.000	32 (28.83)	79 (71.17)	0.059
	postnatal	30 (69.77)	13 (30.23)		14 (32.56)	29 (67.44)		22 (51.16)	21 (48.84)		20 (46.51)	23 (53.49)	
Heart defect correction	complete	16 (66.67)	8 (33.33)	0.268	8 (33.33)	16 (66.67)	0.695	14 (58.33)	10 (41.67)	0.786	10 (41.67)	14 (58.33)	0.152
	incomplete	43 (52.44)	39 (47.56)		28 (34.15)	54 (65.85)		42 (51.22)	40 (48.78)		22 (26.83)	60 (73.17)	
	none	31 (64.58)	17 (35.42)		13 (27.08)	35 (72.92)		24 (50.00)	24 (50.00)		20 (41.67)	28 (58.33)	
PSS-10 categories	Low + average	32 (86.49)	5 (13.51)	<0.001	19 (51.35)	18 (48.65)	0.006	30 (81.08)	7 (18.92)	<0.001	27 (72.97)	10 (27.03)	<0.001
	high	58 (49.57)	59 (50.43)		30 (25.64)	87 (74.36)		50 (42.74)	67 (57.26)		25 (21.37)	92 (78.63)	
Parents’ age	≤29	27 (52.94)	24 (47.06)	0.530	15 (29.41)	36 (70.59)	0.737	18 (35.29)	33 (64.71)	0.013	15 (29.41)	36 (70.59)	0.668
	30–34	31 (58.49)	22 (41.51)		19 (35.85)	34 (64.15)		33 (62.26)	20 (37.74)		20 (37.74)	33 (62.26)	
	3 ≥ 35	32 (64.00)	18 (36.00)		15 (30.00)	35 (70.00)		29 (58.00)	21 (42.00)		17 (34.00)	33 (66.00)	

*p* values were calculated using chi-square test of independence.

**Table 3:** Factors associated with HADS disorders of parents of children with cyanotic congenital heart disease—multivariate logistic regression (N = 154).

		Depression		Anxiety		Aggression/Irritability		Total HADS	
Variable	Category	OR (95%CI)		OR (95%CI)		OR (95%CI)		OR (95%CI)	
		Model 1	Model 2	Model 1	Model 2	Model 1	Model 2	Model 1	Model 2
PSS	low/ medium	1 (ref.)	1 (ref.)	1 (ref.)	1 (ref.)	1 (ref.)	1 (ref.)	1 (ref.)	1 (ref.)
	high	6.91 (2.42; 19.73) *	7.09 (2.42; 20.77) *	3.00 (1.37; 6.57) *	3.12 (1.40; 6.96) *	5.40 (2.15; 13.55) *	5.22 (2.06; 13.21) *	12.02 (4.74; 30.45) *	13.75 (5.09; 37.14) *
Gender	female	1 (ref.)	1 (ref.)	1 (ref.)	1 (ref.)	1 (ref.)	1 (ref.)	1 (ref.)	1 (ref.)
	male	0.39 (0.13; 1.19)	0.39 (0.13; 1.19)	0.50 (0.19; 1.33)	0.50 (0.19; 1.33)	0.97 (0.35; 2.65)	0.96 (0.35; 2.65)	0.42 (0.13; 1.30)	0.41 (0.13; 1.32)
Age	≤29	1 (ref.)	1 (ref.)	1 (ref.)	1 (ref.)	1 (ref.)	1 (ref.)	1 (ref.)	1 (ref.)
	30–34	1.05 (0.44; 2.50)	1.05 (0.44; 2.53)	0.90 (0.38; 2.14)	0.96 (0.40; 2.33)	0.35 (0.15; 0.82) *	0.36 (0.15; 0.86) *	0.92 (0.34; 2.44)	0.87 (0.32; 2.39)
	≥35	0.76 (0.31; 1.83)	0.78 (0.32; 1.90)	1.22 (0.49; 3.00)	1.23 (0.49; 3.03)	0.45 (0.19; 1.06)	0.46 (0.19; 1.09)	1.08 (0.40; 2.97)	1.14 (0.41; 3.19)
Place of residence	other	1 (ref.)	1 (ref.)	1 (ref.)	1 (ref.)	1 (ref.)	1 (ref.)	1 (ref.)	1 (ref.)
	city	2.81 (1.14; 6.94) *	2.81 (1.13; 7.01) *	1.14 (0.49; 2.65)	1.19 (0.51; 2.79)	0.62 (0.27; 1.46)	0.62 (0.27; 1.46)	2.67 (1.07; 6.64) *	2.57 (1.00; 6.55) *
Time of diagnosis	prenatal	1 (ref.)	1 (ref.)	1 (ref.)	1 (ref.)	1 (ref.)	1 (ref.)	1 (ref.)	1 (ref.)
	postnatal	0.44 (0.19; 0.99) *	0.48 (0.21; 1.13)	0.88 (0.40; 1.94)	0.81 (0.36; 1.85)	0.95 (0.44; 2.07)	1.03 (0.46; 2.29)	0.33 (0.14; 0.81) *	0.36 (0.14; 0.90) *
Parents’ education	other	-	1 (ref.)	-	1 (ref.)	-	1 (ref.)	-	1 (ref.)
	higher	-	1.05 (0.50; 2.19)	-	0.99 (0.48; 2.06)	-	1.36 (0.67; 2.76)	-	0.72 (0.31; 1.68)
Heart defect correction	complete	-	1 (ref.)	-	1 (ref.)	-	1 (ref.)	-	1 (ref.)
	incomplete	-	1.84 (0.64; 5.27)	-	0.97 (0.35; 2.68)	-	1.43 (0.53; 3.90)	-	2.06 (0.66; 6.46)
	none	-	1.30 (0.41; 4.06)	-	1.49 (0.49; 4.58)	-	1.37 (0.47; 4.02)	-	1.24 (0.37; 4.13)

Model 1 includes the following independent variables: PSS-10, gender, age, education, place of residence, time of heart disease diagnosis; Model 2 includes the following independent variables: PSS-10, gender, age, education, place of residence, time of heart disease diagnosis and defect correction; OR—odds ratio for occurrence of disorders vs. no disorders + borderline; * *p* < 0.05.

## Data Availability

The data presented in this study are available on request from the corresponding author. The data are not publicly available due to specific ethical and privacy considerations.
